# Factors determining long-term outcomes of hepatocellular carcinoma within the Milan criteria: liver transplantation versus locoregional therapy: A retrospective cohort study

**DOI:** 10.1097/MD.0000000000004735

**Published:** 2016-09-02

**Authors:** Jung Hee Kim, Dong Hyun Sinn, Geum-Youn Gwak, Gyu-Seong Choi, Jong Man Kim, Choon Hyuck David Kwon, Jae-Won Joh, Ki Yeon Kim, Kyunga Kim, Yong-Han Paik, Moon Seok Choi, Joon Hyeok Lee, Kwang Cheol Koh, Seung Woon Paik

**Affiliations:** a Department of Medicine; b Department of Surgery; c Department of Biostatistics and Clinical Epidemiology Center, Samsung Medical Center, Sungkyunkwan University School of Medicine, Korea.

**Keywords:** hepatocellular carcinoma, liver transplantation, locoregional therapy, Milan criteria, survival

## Abstract

Patients with hepatocellular carcinoma (HCC) satisfying the Milan criteria are candidates for liver transplantation (LT), but locoregional therapies could be another options for them.

A total of 1859 treatment-naïve HCC patients fulfilling the Milan criteria were analyzed. Survival tree analysis was performed to generate survival nodes with similar survival risks in 1729 non-LT group, and compared with the survival of 130 patients who received LT.

Among patients who did not receive LT, survival tree analysis classified patients into 6 nodes according to Child-Pugh (CP) score, serum alphafetoprotein (AFP) levels, tumor size, and age, with different mortality risks (5-year survival rate of 87.3%, 77.5%, 65.8%, 64.7%, 44.0%, and 28.7% for nodes 1–6, respectively; *P* < 0.001). The overall survival of patients in nodes 1 (CP score 5 with AFP levels <5 ng/mL) and 2 (CP score 5 with maximal tumor size <2.5 cm) were comparable with that of patients who received LT (both *P* > 0.05), but the survival rates of patients in nodes 3 to 6 were worse than that of LT (*P* < 0.05 for all). In each survival node, survival differed slightly according to initial treatment modality for patients who did not receive LT. For patients who received LT, tumor stage at the time of LT was associated with long-term outcome.

Certain groups of non-LT patients showed survival rates that were similar to the survival rates of LT patients. CP score, AFP levels, tumor size, and age were baseline factors that can help estimate the long-term outcomes of non-LT treatment. In addition, tumor stage at the time of LT and specific initial treatment modality in non-LT patients affected the long-term outcomes. These factors can help estimate the long-term outcomes of HCC patients diagnosed within the Milan criteria.

## 1 Introduction

A landmark study by Mazzaferro et al in 1996^[1]^ established deceased-donor liver transplantation (DDLT) as a valuable option for the treatment of hepatocellular carcinoma (HCC).^[2]^ When liver transplantation (LT) is restricted to patients with early HCC, defined as a single lesion ≤5 cm, up to 3 separate lesions with none >3 cm, with no evidence of gross vascular invasion, and no regional nodal or distant metastases (known as the Milan criteria), a 4-year survival rate of 75% can be achieved.^[1]^ These results have been validated, and with respect to any other available treatment for HCC, LT has the highest potential to cure, as it allows for removal at once of both the tumor and damaged hepatic tissue.^[3]^

However, in most Asian countries, a serious shortage of deceased donors and a strong demand for LT has led to the development of living-donor LT (LDLT) as a practical alternative for DDLT.^[4]^ LDLT is not limited by the restrictions imposed by the nationwide allocation system, and the decision for transplantation often depends on institutional or case-by-case considerations, balancing the will of the donor, the operative risk for both the donor and the recipient, and the overall survival benefit for the recipient.^[5]^ In this respect, an important question arises as to whether there is a survival benefit of LT compared with locoregional therapies in early-stage HCC patients with preserved liver function. Hepatic resection can yield a comparable 5-year survival rate with minimal morbidity compared with LT in patients with early HCC who have adequate liver reserves.^[6]^ Radiofrequency ablation (RFA) is another treatment option for early-stage tumors,^[7,8]^ and RFA for HCC conforming to the Milan criteria showed similar 5-year survival as did surgical resection.^[9]^ Hence, it is still unclear which is better, LT or locoregional therapy in the treatment of HCC that was diagnosed within the Milan criteria, especially if a patient has preserved liver function or if a resection or RFA can be performed.

Therefore, this study was designed to see factors that determine survival of HCC patients diagnosed within the Milan criteria, stratified by those who received LT or who were managed with locoregional therapies. We compared survival rates between patients who received LT and those who did not with the aim of identifying factors that can be used to estimate survival of patients who were diagnosed within the Milan criteria, and did not undergo LT. These factors can help decide between LT versus locoregional therapy in cases diagnosed within the Milan criteria.

## 2 Methods

### 2.1 Study population

The HCC registry of Samsung Medical Center, Seoul, Korea, which enrolls treatment-naïve, newly diagnosed HCC patients who received care at Samsung Medical Center, Seoul, Korea, was used for this study. The registry began in January 2005. When patients are newly diagnosed with HCC, well-trained abstractors collect data, including age at diagnosis, sex, date of diagnosis, etiology, liver function (e.g., Child-Pugh [CP] class), tumor characteristics (e.g., number of tumors, maximal tumor size, the presence and extent of portal vein invasion, and type of extrahepatic spread), tumor stage, and initial treatment modality. HCC was diagnosed either histologically or clinically according to regional guidelines of HCC.^[10,11]^ Among a total of 3515 patients who were registered in the HCC registry between January 1, 2005 and December 31, 2009, we enrolled 1859 HCC patients fulfilling the Milan criteria at the time of HCC diagnosis. This study was reviewed and approved by the Institutional Review Board of Samsung Medical Center. Because the study is based on a retrospective analysis of existing administrative and clinical data, the requirement for obtaining informed patient consent was waived by the Institutional Review Board.

### 2.2 Variables, primary endpoint, and follow-up

Data on each patient included age, sex, etiology of liver disease, serum alphafetoprotein (AFP) levels, model for end-stage liver disease (MELD) score, Eastern Cooperative Oncology Group performance status (ECOG), CP class with score, tumor size, number, extent, and the presence of vessel invasion at the time of HCC diagnosis. Initial treatment modality for HCC was also recorded. The primary endpoint was overall survival, which was defined as the time from the primary diagnosis of HCC to death. All patients were followed up from baseline to June 2015. Patient survival data were collected from the National Statistics Service; therefore, all deaths at the time of survival assessment were certified. We also collected information regarding cause of death using the International Classification of Disease code that was recorded at death certificate of each patient. Liver-related death was defined when the cause of death was related to HCC or liver cirrhosis. For those who received curative treatment (LT, resection, or ablation), information on recurrence was collected.

### 2.3 Statistical analyses

Survival curves were estimated using the Kaplan–Meier method and compared using the log-rank test. Cox-regression analysis was conducted to compare overall survival between groups, adjusted for age. Age, sex, CP score, MELD score, aspartate to platelet ratio index, previous liver decompensation history, tumor number, tumor size, serum AFP levels, ECOG, and underlying liver disease were tested by random survival forest analysis. Decision tree analysis was conducted to detect survival nodes with similar survival risk using R 3.1.0 (Vienna, Austria).

## 3 Results

### 3.1 Baseline characteristics, treatment modalities, and survival

The baseline characteristics of the 1859 HCC patients fulfilling the Milan criteria are shown in Table 1. The mean age of the patients was 57.3 years, and most were male (75.9%) with good performance (ECOG 0, 92.6%) and well-preserved liver function (CP score A, 85.1%). Median AFP level was 23.2 ng/mL. During follow-up, 130 patients received LT (7.0%); their characteristics are shown in Table 2. LDLT was major form (106 patients, 81.5%) and median time from diagnosis to LT was 18.3 months. Thirty-four patients (26.2%) received LT without other therapy and 96 patients (73.8%) received one or more locoregional therapies before LT. At the time of LT, 113 patients (86.9%) were still within the Milan criteria (32 patients who received LT without other therapy and 81 patients who received LT following other therapies) and 17 patients (13.1%) had surpassed the Milan criteria (2 patients who received LT without other therapy and 15 patients who received LT following other therapies). Among 1729 patients who did not undergo LT, the major initial treatment modality was resection (n = 608, 35.2%) followed by RFA (n = 598, 34.5%) and transcatheter arterial chemoembolization (TACE) (n = 510, 29.5%). The median follow-up duration was 72.5 months (range: 0.4–122.9 months). The median survival had not been reached at the time of analysis, and the 5-year survival rate was 68.4%. Among 679 patients who died, 92.2% were liver-related mortality. HCC recurrence was observed in 10.8% (14/130), 43.1% (262/608), and 66.4% (397/598) of patients who underwent LT, resection, and RFA, respectively.

**Table 1:**
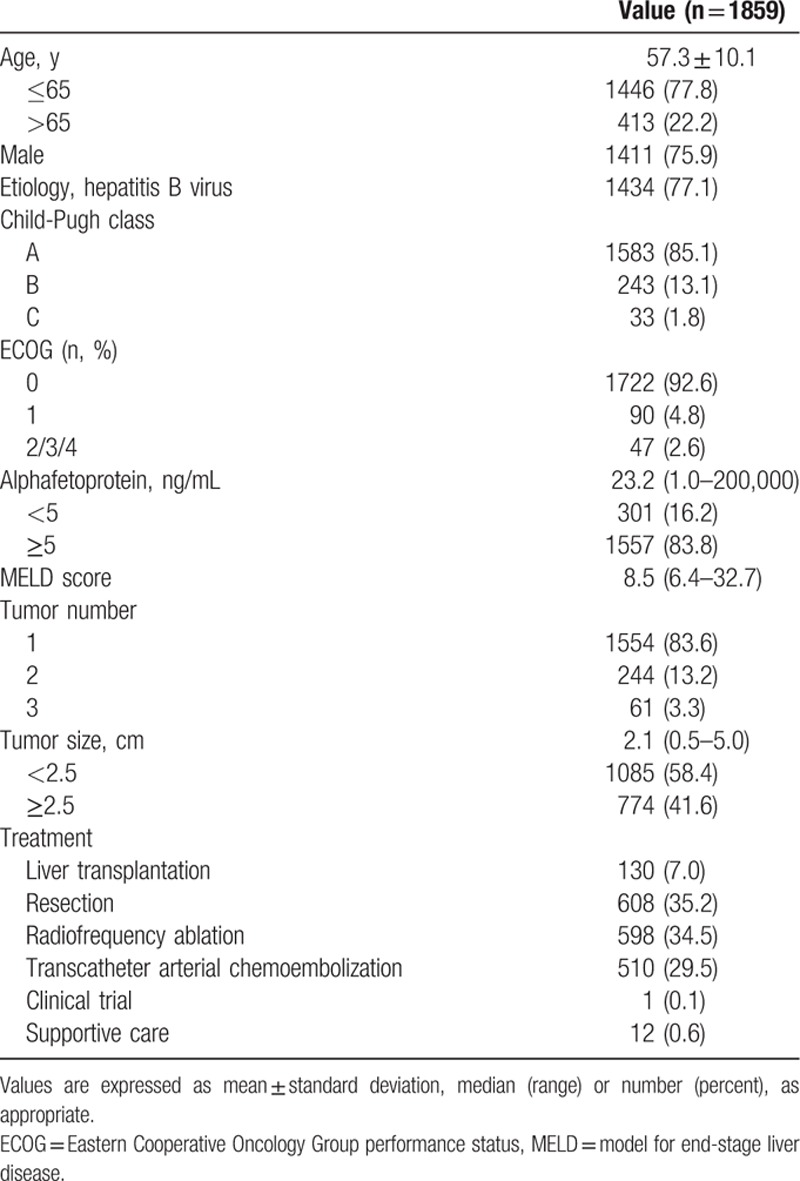
Baseline characteristics.

**Table 2:**
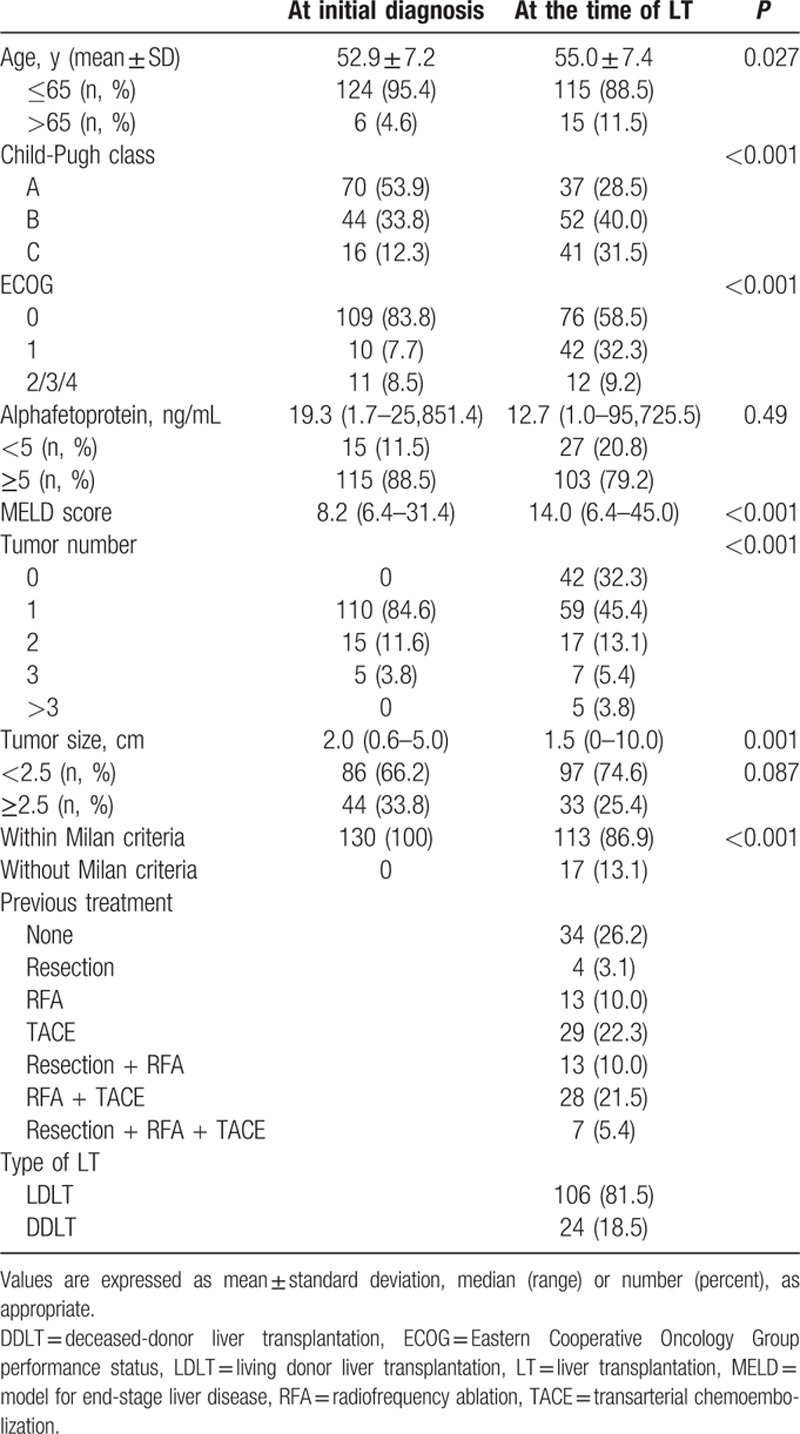
Characteristics of patients who received liver transplantation.

### 3.2 Survival nodes with similar survival in the non-LT group

**Figure 1:**
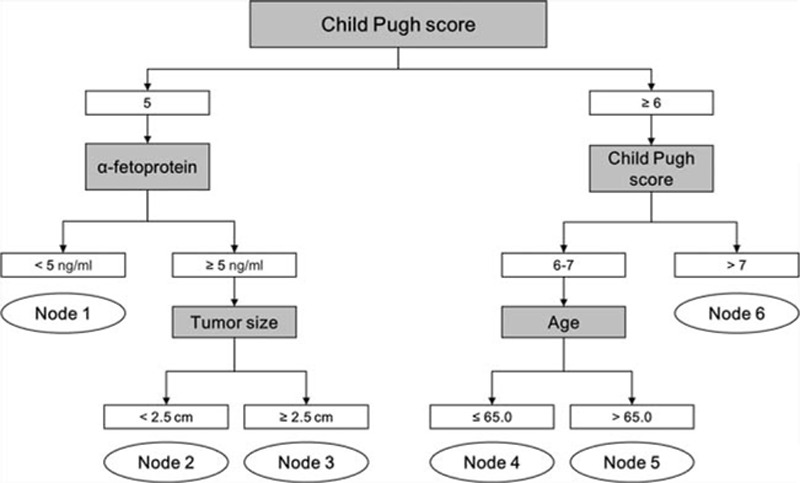
Survival tree analysis of hepatocellular carcinoma patients who did not undergo liver transplantation. Child-Pugh score, serum alphafetoprotein, tumor size, and age were factors that divide the survival tree.

**Figure 2:**
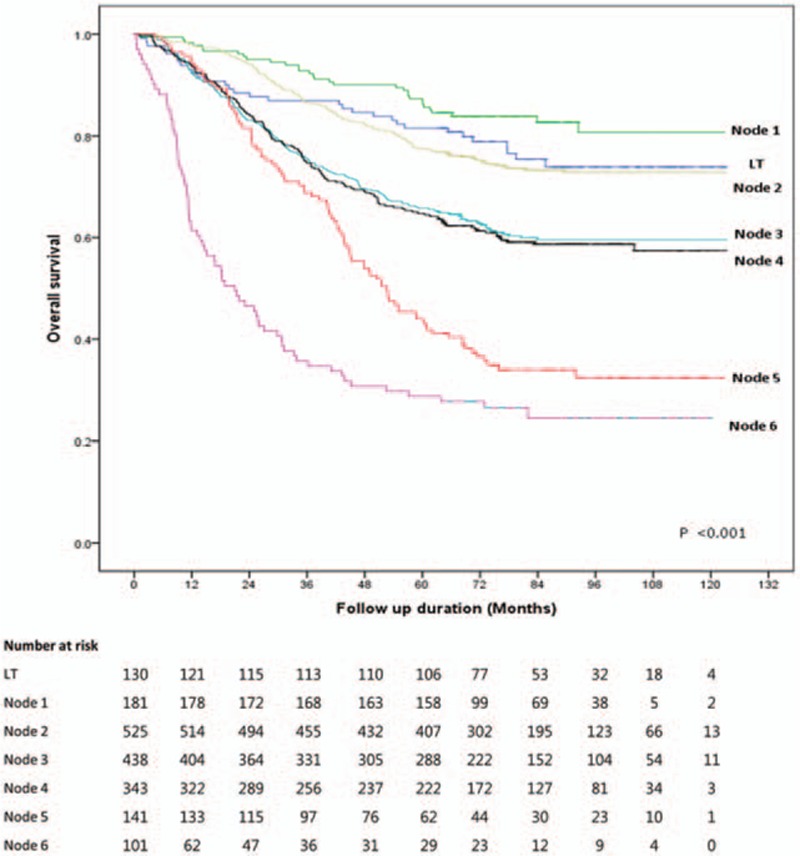
Survival of patients. There were significant differences in overall survival rate by survival node and liver transplantation.

By survival tree analysis, patients in the non-LT group were divided into 6 subgroups (nodes 1–6) with similar survival risks based on CP score, serum AFP level, tumor size, and age (Fig. 1). Node 1 was characterized by CP score 5 and serum AFP <5 ng/mL. Node 2 was characterized by CP score 5, AFP ≥5 ng/mL, and tumor size <2.5 cm. Node 3 was characterized by CP score 5, AFP ≥5 ng/mL, and tumor size ≥2.5 cm. Node 4 was characterized by CP score 6 to 7 and age ≤65 years. Node 5 was characterized by CP score 6 to 7 and age >65 years. Node 6 was characterized by CP score ≥8. The 5-year survival rates of patients in nodes 1, 2, 3, 4, 5, and 6 were 87.3%, 77.5%, 65.8%, 64.7%, 44.0%, and 28.7%, respectively (Fig. 2; Table 3). Liver-related mortality was observed in 83.9%, 90.5%, 93.6%, 96.4%, 90.3%, and 97.3% of patients in nodes 1, 2, 3, 4, 5, and 6, respectively (*P* = 0.048).

**Table 3:**
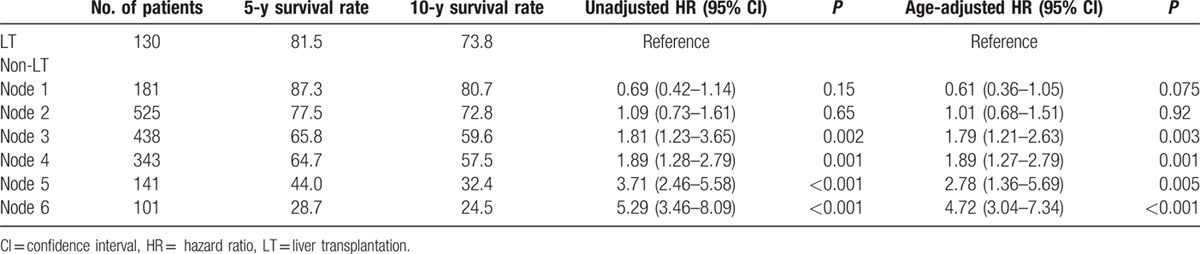
Comparison of survival between each survival node and liver transplantation.

Initial treatment modality was also associated with survival. Overall, the 5-year survival rates were 82.6%, 70.9%, and 46.5% for patients who underwent resection, RFA, and TACE, respectively (*P* < 0.001). Among 1336 patients who received curative therapy (either LT, resection, or RFA), the recurrence rates were 10.8%, 37.8%, 57.4%, 52.5%, 65.4%, 65.0%, and 26.3% for LT, and nodes 1, 2, 3, 4, 5, and 6, respectively (*P* < 0.001). The overall- and recurrence-free survival rate differed according to the initial treatment modality in each survival node (Table 4).

**Table 4:**
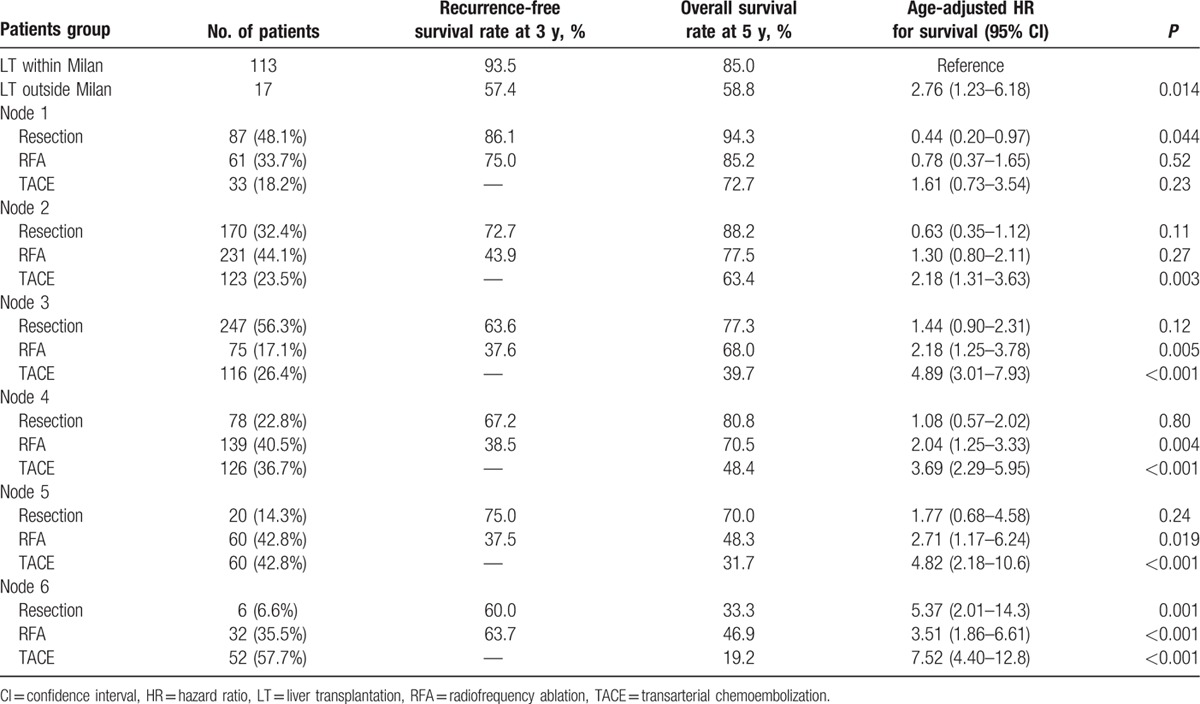
Comparison of survival between treatment modality of each survival node and liver transplantation.

### 3.3 Overall survival according to LT

Among 130 patients with LT, 31 cases of mortality were observed. The liver-related mortality rate was 74.2% (23/31 patients). Among 8 patients with nonliver-related mortality, infectious complication was the most frequent cause of death (4 cases). Liver-related mortality was less frequently observed in those who received LT than in those who received non-LT treatment (74.2% vs 93.1%, *P* < 0.001).

Overall survival was longer in patients who received LT (81.5% vs 67.4% at 5 years; 73.8% vs 61.1% at 10 years, *P* = 0.002). The unadjusted hazard ratio (HR) for overall survival (LT vs non-LT) was 0.58 (95% confidence interval [CI], 0.40–0.83) (*P* = 0.003). LT remained a significant factor for overall survival after adjustment for age, sex, CP class, tumor number, tumor size, serum AFP level, and ECOG status (HR = 0.46; 95% CI, 0.32–0.67, *P* < 0.001).

When patients who received LT were classified according to the same criteria used to categorize non-LT patients, there were no significant differences in survival by node; the 5-year survival rates were 83.3%, 78.6%, 84.6%, 81.1%, 75.0%, and 82.5% for nodes 1 (n = 6), 2 (n = 14), 3 (n = 13), 4 (n = 53), 5 (n = 4), and 6 (n = 40), respectively (*P* = 0.76). Therefore, when we compared survival according to LT in each node, the survival of all LT patients was used as a reference instead of that of LT patients in the corresponding node (Table 3).

**Figure 3:**
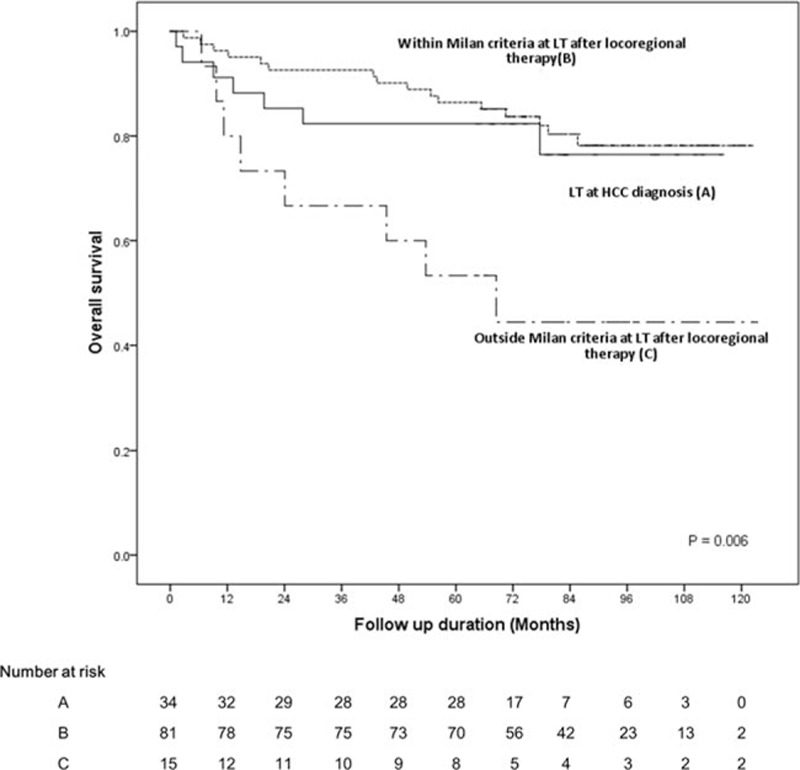
Survival of hepatocellular carcinoma patients who received liver transplantation. Patient survival significantly decreased when the tumor had surpassed the Milan criteria (stage migration) at the time of liver transplantation.

When stratified according to the purpose of LT (primary vs salvage) and tumor stage at the time of LT (within vs beyond the Milan criteria), the 5-year survival rate was 82.4% for patients who underwent primary LT (n = 34), 86.4% for patients who received locoregional therapies first and then salvage LT and who met the Milan criteria at the time of LT (n = 81), and 53.3% for patients who received locoregional therapies first and then salvage LT who had surpassed the Milan criteria (n = 15) (Fig. 3). Two patients who received primary LT had stage migration at the time of LT. Baseline characteristics of patients who received LT within and beyond the Milan criteria were similar, except for tumor size (median: 1.8 vs 2.8 cm, *P* = 0.004). Overall survival and recurrence-free survival were better for those who received LT and met the Milan criteria compared with those who received LT who surpassed the Milan criteria at the time of LT (Table 4).

### 3.4 Comparison of survival between LT versus each survival node (non-LT)

The overall survival rates of patients in nodes 1 and 2 in the non-LT group were similar to that in the LT group; however, the survival of patients in nodes 3, 4, 5, and 6 in the non-LT group were significantly worse (Fig. 2). Unadjusted and age-adjusted HR for survival of each node (non-LT) vs LT was comparable in nodes 1 and 2, but significantly worse in nodes 3, 4, 5, and 6 (Table 3). For patients who received LT, tumor stage at the time of LT was associated with overall survival. For patients who received non-LT treatment, initial treatment modality was associated with patient survival. Thus, when each survival node was further stratified by initial treatment modality, resection produced even better survival than LT in node 1, similar survival in nodes 2, 3, 4, and 5, and worse survival in node 6 compared with patients who received LT within the Milan criteria (Table 4). RFA showed similar survival to LT in nodes 1 and 2, and showed worse survival in nodes 3, 4, 5, and 6. TACE showed similar survival to LT only in node 1, and showed worse survival in the other survival nodes.

## 4 Discussion

In this study, the first factor that divided the survival probability of patients diagnosed within the Milan criteria who did not undergo LT was CP score. Those with CP score ≥8 (node 6) showed the worst 5-year survival rate (28.7%). Among patients with CP score 6 to 7, age was the next factor that classified patient survival risk (nodes 4 and 5). In patients with a CP score of 5, AFP and tumor size were the next factors that further classified patients (nodes 1, 2, and 3). This survival tree analysis clearly demonstrated that for patients with decreased liver function, defined by CP score ≥6 (nodes 4, 5, and 6), the best treatment option is LT in terms of long-term survival. In case with preserved liver function (CP score 5), our data indicate that serum AFP and tumor size may help identify the subset of patients who might achieve survival benefit by LT. Node 1, which is characterized by preserved liver function (CP score 5) plus extremely low AFP levels (<5 ng/mL), and node 2, which is characterized by preserved liver function (CP score 5) plus small tumor (size <2.5 cm), showed similar survival to LT, whereas node 3, characterized by preserved liver function (CP score 5) but AFP ≥5 ng/mL and tumor size ≥2.5 cm), showed a lower survival rate than LT, indicating that LT is the best option for node 3, whereas locoregional therapies may be preferable in patients in nodes 1 and 2.

The reason for the better survival following LT in patients with decreased liver function (nodes 4 to 6) can be explained because liver function is a well-known factor for patient survival in HCC,^[12,13]^ and LT can treat both the tumor and the damaged hepatic tissue.^[3,4]^ In patients with preserved liver function, LT provided a survival benefit compared with locoregional therapies in survival node 3, but not in nodes 1 and 2. AFP level is an important factor for prognosis in HCC.^[14–16]^ Tumor size is also an important prognostic factor.^[17,18]^ Larger tumor size is an important risk factor for local tumor progression after RFA^[7]^; it decreases the effect of locoregional treatment and increases the recurrence rate compared with smaller sized tumors.^[19,20]^ This data shows that similar long-term survival can be expected following non-LT treatment for patients with preserved liver function plus low AFP levels (node 1) or preserved liver function and small tumors (node 2).

For those who received LT, tumor stage at LT was associated with long-term outcome. Notably, only 34 patients underwent primary LT, with the remainder receiving one or more locoregional therapies to control HCC before LT (salvage LT). It is noteworthy that those who were still within the Milan criteria at the time of LT (86.4% at 5 years) displayed similar survival to those who underwent primary LT (82.4% at 5 years). Hence, as long as a patient stays within the Milan criteria, salvage LT, that is, liver resection (or other locoregional therapies) for HCC as a first-line treatment in transplantable patients followed by transplantation, is an attractive option.^[21]^ Resection has been reported to be more cost-effective than LT for early HCC within the Milan criteria,^[22]^ and performing LT too soon after diagnosis was suggested as a factor that adversely affect patient outcomes.^[23]^ Similar outcomes after LT have also been reported between primary versus salvage LDLT.^[24]^ In our series, 13.1% (17/130) of patients were transplanted after their tumor had progressed beyond the Milan criteria; those patients showed a significantly lower survival rate (53.3% at 5 years). Fuks et al also reported that 22% of transplantable patients became nontransplantable as the disease progressed beyond the Milan criteria when treated with logoregional therapies.^[25]^ Microscopic vascular invasion, satellite nodules, and tumor size >3 cm poorly differentiated tumors, and liver cirrhosis are risk factors for the recurrence beyond Milan criteria.^[25]^ In our analysis, large tumor size was associated with LT beyond the Milan criteria. When patients start therapy with non-LT treatment, there is a risk of stage migration (recurrence beyond the Milan criteria). Hence, while considering the salvage LDLT, one should be aware of the risk of stage migration following non-LT treatment, and the risk should be discussed carefully with the patient.

For those who did not receive LT, treatment modality was associated with long-term outcome. Resection showed better survival than RFA or TACE in each survival node. Thus, when compared with LT within the Milan criteria (Table 4), resection showed even better survival than LT in node 1, similar survival in nodes 2, 3, 4, and 5, and worse survival in node 6. RFA showed similar survival to LT in nodes 1 and 2, and worse survival in nodes 3, 4, 5, and 6. TACE showed similar survival to LT only in node 1. Generally, patients who receive resection are carefully selected and have adequate liver functional reserves,^[11]^ which explains why patients with resection showed better survival in each survival node. Yet, this finding illustrates that initial treatment modality can also affect long-term outcome. Hence, when estimating long-term outcome, one must consider the available treatment options for each patient. For example, similar long-term outcome to LT can be expected by resection or RFA for patients in node 2, but not with TACE. Likewise, similar long-term outcome to LT can be expected if resection can be done in survival node 3.

There are some limitations to this study. The retrospective design is an inherent limitation. Our survival tree analysis was based on baseline characteristics. However, several on-treatment factors can affect survival of HCC patients, such as treatment response, AFP levels after treatment, tumor recurrence, and subsequent treatment.^[13]^ Likewise, recurrence of underlying disease (e.g., hepatitis C) and recurrence of tumor can affect long-term outcomes in patients who received LT.^[26]^ HCC is notorious for its high recurrence rate, and treatment of recurrence affects long-term outcome,^[27]^ yet this study analyzed the initial treatment only. More importantly, selection of the treatment modality was chosen by a respective physician. There is thus a selection bias regarding the choice of each treatment, and also there may be an unidentified or unrecorded bias. Tumor recurrence, cost, quality of life, and donor availability are other issues that need to be considered when selecting treatment modality. The study was conducted in a deceased donor resource-poor country, where LDLT is the major form of LT. The strength of this study is that it included a large number of HCC patients and that there was no follow-up loss in the survival statistics.

In summary, this study identified important determinants for survival that can be used to estimate survival of patients diagnosed within the Milan criteria, which can help doctors in choosing a first-line treatment option. Our data indicate that LT should be the first-line option for those with decreased liver function. For patients with preserved liver function, those with low serum AFP levels or small tumors showed comparable survival to LT, indicating that these 2 factors can be used to estimate survival of non-LT patients. For patients who received LT, baseline factors were not significantly linked to survival, yet tumor stage at the time of LT was associated with long-term outcome, indicating that when choosing locoregional therapy versus LT, one must consider the risk of tumor progression beyond the Milan criteria when managed with locoregional therapies. For patients receiving non-LT treatment, availability of a specific treatment modality (resection, RFA or TACE) also affected long-term outcome. In conclusion, CP score, serum AFP levels, tumor size, and age are baseline factors that can be used to estimate long-term outcomes in non-LT patients. Tumor progression beyond the Milan criteria and availability of specific treatment modalities also affected long-term outcomes. These factors may be used to estimate long-term outcomes of HCC patients diagnosed within the Milan criteria.

## References

[1] Mazzaferro V Regalia E Doci R Liver transplantation for the treatment of small hepatocellular carcinomas in patients with cirrhosis. *N Engl J Med* 1996; 334:693–699. 8594428 10.1056/NEJM199603143341104

[2] Poon D Anderson BO Chen LT Management of hepatocellular carcinoma in Asia: consensus statement from the Asian Oncology Summit 2009. *Lancet Oncol* 2009; 10:1111–1118. 19880065 10.1016/S1470-2045(09)70241-4

[3] Mancuso A Perricone G Hepatocellular carcinoma and liver transplantation: state of the art. *J Clin Transl Hepatol* 2014; 2:176–181. 26357625 10.14218/JCTH.2014.00013 PMC4521243

[4] Hwang S Lee SG Belghiti J Liver transplantation for HCC: its role: Eastern and Western perspectives. *J Hepatobiliary Pancreat Sci* 2010; 17:443–448. 19885638 10.1007/s00534-009-0241-0

[5] Tamura S Sugawara Y Kokudo N Living donor liver transplantation for hepatocellular carcinoma: the Japanese experience. *Oncology* 2011; 81 suppl 1:111–115. 22212944 10.1159/000333270

[6] Cha CH Ruo L Fong Y Resection of hepatocellular carcinoma in patients otherwise eligible for transplantation. *Ann Surg* 2003; 238:315–321. 14501497 10.1097/01.sla.0000086548.84705.ef PMC1422705

[7] Kim YS Lim HK Rhim H Ten-year outcomes of percutaneous radiofrequency ablation as first-line therapy of early hepatocellular carcinoma: analysis of prognostic factors. *J Hepatol* 2013; 58:89–97. 23023009 10.1016/j.jhep.2012.09.020

[8] Rhim H Lim HK Radiofrequency ablation of hepatocellular carcinoma: pros and cons. *Gut Liver* 2010; 4 suppl 1:S113–S118. 21103289 10.5009/gnl.2010.4.S1.S113 PMC2989542

[9] Yi HM Zhang W Ai X Radiofrequency ablation versus surgical resection for the treatment of hepatocellular carcinoma conforming to the Milan criteria: systemic review and meta-analysis. *Int J Clin Exp Med* 2014; 7:3150–3163. 25419346 PMC4238508

[10] Korean Liver Cancer Study Group, National Cancer Center. [Practice guidelines for management of hepatocellular carcinoma 2009]. *Korean J Hepatol* 2009; 15:391–423. 19783891 10.3350/kjhep.2009.15.3.391

[11] Korean Liver Cancer Study Group, National Cancer Center, Korea. 2014 KLCSG-NCC Korea Practice Guideline for the Management of Hepatocellular Carcinoma. *Gut Liver* 2015; 9:267–317. 25918260 10.5009/gnl14460 PMC4413964

[12] Forner A Llovet JM Bruix J Hepatocellular carcinoma. *Lancet* 2012; 379:1245–1255. 22353262 10.1016/S0140-6736(11)61347-0 PMC13537732

[13] Au JS Frenette CT Management of hepatocellular carcinoma: current status and future directions. *Gut Liver* 2015; 9:437–448. 26087860 10.5009/gnl15022 PMC4477987

[14] Farinati F Marino D De Giorgio M Diagnostic and prognostic role of alpha-fetoprotein in hepatocellular carcinoma: both or neither? *Am J Gastroenterol* 2006; 101:524–532. 16542289 10.1111/j.1572-0241.2006.00443.x

[15] Nomura F Ohnishi K Tanabe Y Clinical features and prognosis of hepatocellular carcinoma with reference to serum alpha-fetoprotein levels. Analysis of 606 patients. *Cancer* 1989; 64:1700–1707. 2477133 10.1002/1097-0142(19891015)64:8<1700::aid-cncr2820640824>3.0.co;2-z

[16] Suh SW Lee KW Lee JM Prediction of aggressiveness in early-stage hepatocellular carcinoma for selection of surgical resection. *J Hepatol* 2014; 60:1219–1224. 24548529 10.1016/j.jhep.2014.01.027

[17] Zhang W Wang X Jiang R Effect of tumor size on cancer-specific survival in small hepatocellular carcinoma. *Mayo Clin Proc* 2015; 90:1187–1195. 26231292 10.1016/j.mayocp.2015.06.018

[18] Chen YL Ko CJ Chien SY Tumor size as a prognostic factor in resected small hepatocellular carcinoma: a controversy revisited. *J Gastroenterol Hepatol* 2011; 26:851–857. 21129015 10.1111/j.1440-1746.2010.06595.x

[19] Pawlik TM Delman KA Vauthey JN Tumor size predicts vascular invasion and histologic grade: implications for selection of surgical treatment for hepatocellular carcinoma. *Liver Transpl* 2005; 11:1086–1092. 16123959 10.1002/lt.20472

[20] Tabrizian P Jibara G Shrager B Recurrence of hepatocellular cancer after resection: patterns, treatments, and prognosis. *Ann Surg* 2015; 261:947–955. 25010665 10.1097/SLA.0000000000000710

[21] Chan DL Alzahrani NA Morris DL Systematic review of efficacy and outcomes of salvage liver transplantation after primary hepatic resection for hepatocellular carcinoma. *J Gastroenterol Hepatol* 2014; 29:31–41. 24117517 10.1111/jgh.12399

[22] Lim KC Wang VW Siddiqui FJ Cost-effectiveness analysis of liver resection versus transplantation for early hepatocellular carcinoma within the Milan criteria. *Hepatology* 2015; 61:227–237. 24638991 10.1002/hep.27135

[23] Halazun KJ Patzer RE Rana AA Standing the test of time: outcomes of a decade of prioritizing patients with hepatocellular carcinoma, results of the UNOS natural geographic experiment. *Hepatology* 2014; 60:1957–1962. 24954365 10.1002/hep.27272

[24] Moon JI Kwon CH Joh JW Primary versus salvage living donor liver transplantation for patients with hepatocellular carcinoma: impact of microvascular invasion on survival. *Transplant Proc* 2012; 44:487–493. 22410053 10.1016/j.transproceed.2011.11.009

[25] Fuks D Dokmak S Paradis V Benefit of initial resection of hepatocellular carcinoma followed by transplantation in case of recurrence: an intention-to-treat analysis. *Hepatology* 2012; 55:132–140. 21932387 10.1002/hep.24680

[26] Lucey MR Terrault N Ojo L Long-term management of the successful adult liver transplant: 2012 practice guideline by the American Association for the Study of Liver Diseases and the American Society of Transplantation. *Liver Transpl* 2013; 19:3–26. 23281277 10.1002/lt.23566

[27] Yu SJ A concise review of updated guidelines regarding the management of hepatocellular carcinoma around the world: 2010–2016. *Clin Mol Hepatol* 2016; 22:7–17. 27044761 10.3350/cmh.2016.22.1.7 PMC4825164

